# Dietary Protein in Older Adults: Adequate Daily Intake but Potential for Improved Distribution

**DOI:** 10.3390/nu9030184

**Published:** 2017-02-23

**Authors:** Danielle K. Cardon-Thomas, Timothy Riviere, Zoë Tieges, Carolyn A. Greig

**Affiliations:** 1 School of Sport, Exercise and Rehabilitation Sciences, University of Birmingham, Birmingham B15 2TT, UK; dxt353@bham.ac.uk (D.K.C.-T.); tim.riviere@googlemail.com (T.R.); 2 MRC Arthritis Research UK Centre for Musculoskeletal Ageing Research, Birmingham B15 2TT, UK; 3 Geriatric Medicine, University of Edinburgh, Edinburgh EH16 4SA, UK; zoe.tieges@ed.ac.uk

**Keywords:** protein intake, protein distribution, ageing, sarcopenia, physical activity, sedentary behavior

## Abstract

Daily distribution of dietary protein may be important in protecting against sarcopenia, specifically in terms of per meal amounts relative to a proposed threshold for maximal response. The aims of this study were to determine total and per meal protein intake in older adults, as well as identifying associations with physical activity and sedentary behavior. Three-day food diaries recorded protein intake in 38 participants. Protein distribution, coefficient of variation (CV), and per meal amounts were calculated. Accelerometry was used to collect physical activity data as well as volume and patterns of sedentary time. Average intake was 1.14 g·kg^−1^·day^−1^. Distribution was uneven (CV = 0.67), and 79% of participants reported <0.4 g·kg^−1^ protein content in at least 2/3 daily meals. Protein intake was significantly correlated with step count (*r* = 0.439, *p* = 0.007) and negatively correlated with sedentary time (*r* = −0.456, *p* = 0.005) and Gini index G, which describes the pattern of accumulation of sedentary time (*r* = −0.421, *p* = 0.011). Total daily protein intake was sufficient; however, distribution did not align with the current literature; increasing protein intake may help to facilitate optimization of distribution. Associations between protein and other risk factors for sarcopenia may also inform protective strategies.

## 1. Introduction

Ageing is associated with a decline in skeletal muscle mass and function (sarcopenia), which impacts adversely upon musculoskeletal health and physical independence [1]. Modification of lifestyle factors may be useful tools to combat sarcopenia. Protein ingestion elicits a muscle protein synthetic (MPS) response; however, this acute anabolic response is blunted in older muscle [2]. Along with muscle protein breakdown, MPS contributes to net protein accretion over time; therefore, blunted MPS can translate into chronic maladaptation in older adults [3]. Hence, optimization of dietary protein to maximize MPS has received a great deal of interest. The current Recommended Daily Allowance (RDA) for protein is 0.8–1.2 g·kg^−1^·day^−1^ for both younger and older adults [4]; however, this is contentious, and recent recommendations propose an increased lower limit of 1.0 g·kg^−1^·day^−1^ [5].

There is growing evidence to support the theory that the distribution of protein across the day can affect anabolism [6,7]. This is based on the relationship between protein ingestion and the acute MPS response, which is dose-dependent up to a threshold, beyond which MPS plateaus and there is no increase with larger amounts of protein [2,8]. Hence, distributing protein evenly across daily meals to reach this threshold multiple times may stimulate greater MPS across the day than a more skewed distribution. Indeed, a recent study investigating protein intake in men and women aged 50–85 years reported greater lean mass and muscle strength in participants consuming a higher frequency of meals containing at least 30 g of protein [9]. However, this study and others investigating the protein dose threshold for maximal MPS have expressed protein as an absolute value, whereas intake recommendations are given relative to body weight. A retrospective analysis of data from older men (mean 71 years), which included MPS responses to a range of protein doses, has identified a relative proposed threshold to elicit maximal MPS of 0.4 g·kg^−1^ [8].

In addition to dietary protein, low physical activity has been identified as a risk factor for sarcopenia by association with skeletal muscle mass index [10,11]. Relatively low participation in physical activity among older adults [12] indicates another potential target for intervention. Distinct from this is sedentary behavior, defined as non-exercise (low energy expenditure) seated or lying behavior, which is also a risk factor for a number of health issues including sarcopenia [13], as reflected in public health guidelines, which recommend minimization of sitting time [14]. As with protein intake, the pattern of accumulation as well as the volume of sedentary time is important; evidence indicates benefits of breaking up sedentary bouts with periods of activity [15]. Along with dietary protein, these are modifiable lifestyle factors that can influence skeletal muscle in older age, and the identification of associations between these factors may be informative.

The Recommended Daily Allowance does not currently specify a recommendation for per meal protein amount. As data relating to dietary protein distribution and how dietary habits align with the literature is lacking, it is not clear whether interventions are required to optimize protein distribution. The design of interventions and recommendations is therefore limited. The aim of this study was to determine the total and per meal protein intake in a group of older adults, with a secondary aim to investigate associations between protein intake and active and sedentary behaviors.

## 2. Materials and Methods 

### 2.1. Study Population

Community dwelling older adults were recruited from July to November 2014, through local housing trusts and a database of volunteers. To be included, participants needed to be ≥70 years of age, ambulatory, and living independently. Participant characteristics including age, sex, and estimated body weight were collected. This study was conducted in accordance with the Declaration of Helsinki and was approved by a local ethics committee (27 March 2014, ERN_13-1475).

### 2.2. Nutritional Data Collection

Dietary intake was recorded using three-day food diaries, completed over three consecutive days including at least one weekend day. Participants were given verbal and written instructions to record details and timing of all food and drink consumed, with amounts weighed where appropriate or estimated based on written portion guide instructions. A follow-up meeting was conducted where participants gave verbal clarification where required.

### 2.3. Physical Activity Data Collection

Objective measurement of physical activity and sedentary behavior was obtained using ActivPAL3™ accelerometers, worn for seven days. Monitors were attached to the anterior of the thigh, and participants were instructed to remove only for swimming and bathing.

### 2.4. Nutritional Data Analysis

Food diary data were entered into Dietplan6 software (Forestfield Ltd., West Sussex, UK, v6.70.73), which generated energy, macronutrient and micronutrient results. Protein values were calculated relative to body weight (g·kg^−1^), as well as energy from protein as a percentage of total energy intake. Proportions of protein originating from plant and animal sources were extracted. To determine protein distribution, days were split into 30 min time slots, with intake within each slot considered an individual eating episode. Days were then divided into three time periods, 05:00–11:00 (period 1), 11:00–16:00 (period 2) and 16:00–23:59 (period 3), to encompass breakfast, lunch and dinner respectively. A fourth time period was initially considered to represent an afternoon snack; however, this was rejected due to negligible protein content. Average protein intake for each 30 min slot was calculated, and summed for each time period to give average per meal intake. As the main meal of the day, defined as the meal with the highest protein intake, varied between lunch and dinner, the main mealtime was identified for each participant.

Average protein intakes were calculated for each time period to determine whether protein intake was skewed or evenly distributed across the day. This was quantified by calculating the coefficient of variance (CV) of protein distribution, a dimensionless measure of distribution (CV = standard deviation/mean), which indicates evenness of intake across meals; a CV of zero would indicate the same amount in each period [16]. The pattern of intake was also analyzed by assigning days to categories of distribution depending on the relative differences in protein intakes between meals. Each day was categorized separately, assuming the maximum amount in each time period represented the main meal in that period, and a difference between meals was defined by a threshold of 0.1 g·kg^−1^ (approximately 10% of total intake). Nine distinct categories were generated.

Per meal protein intake was analyzed relative to the proposed 0.4 g·kg^−1^ threshold for maximal MPS [8]. The proportion of meals that failed to reach this threshold was calculated for each time period, as well as the number of meals per day which were below the threshold for each participant (range 0–3).

### 2.5. Physical Activity and Sedentary Data Analysis

Activity data were downloaded from monitors using ActivPAL™ Analysis software (PAL Technologies Ltd., Glasgow, UK, v7.2.29). Only data from fully recorded days were used, which gave an average of 6 days per participant, and no attempt was made to remove sleep time (day or night) from sedentary data. Average daily step count, and volume of sedentary, standing and stepping time were extracted.

Sedentary data from activity monitors were processed using MATLAB (The MathWorks Inc., Natick, MA, USA, vR2012b) [17] to calculate variables to describe the patterns of sedentary behavior: (a) Weighted median sedentary bout length: length of the sedentary bout that corresponded to 50% of accumulated sedentary time in each participant. Higher values indicate that sedentary time was accumulated predominantly in longer sedentary bouts [18]; (b) Inter-sitting time: average difference between the start times of each two consecutive sedentary bouts, high values indicate either infrequent sitting down from standing or activity, or long periods of sitting with few transitions to standing; (c) Fragmentation index: ratio of number of sedentary bouts to total sedentary time; high values indicate accumulation of sedentary time in short, frequent bouts as opposed to longer, infrequent bouts [18]; (d) Gini index G: a standardized statistic, range 0–1, calculated by integration of the Lorentz curve [17]. A value of zero indicates that all sedentary bouts lengths contribute equally to sedentary time (i.e., a fragmented pattern), while a value close to one indicates accumulation in a small proportion of long bouts.

### 2.6. Statistical Analysis

Sample size was determined by point estimate calculations for protein intake to allow a margin for random error of 0.09 g·kg^−1^·day^−1^. Data were assessed using SPSS Statistics (IBM, New York, NY, USA, v23). Data are reported as mean (standard deviation). *t*-tests were used to identify differences between men and women, Pearson’s correlation coefficients were calculated to assess associations between variables, and one-way ANOVAs were used to identify differences when participants were grouped by the number of daily meals containing less than 0.4 g·kg^−1^ of protein. Where assumptions of normality were not met, non-parametric tests were used. All tests were completed to a 95% significance level, and data are reported as mean (SD).

## 3. Results

Thirty-eight participants took part in the study (12 men, 26 women), and of these 36 provided a complete data set; one participant opted not to wear the activity monitor, and for another there were technical issues when extracting data from the monitor. Participant characteristics are displayed in Table 1, along with key variables for protein intake and activity data. No differences were found between men and women for any variable. All variables were normally distributed except percentage of energy from protein, step count, bout length and fragmentation index.

### 3.1. Protein Intake

Average protein intake was 1.14 (0.25) g·kg^−1^·day^−1^. This was not significantly correlated with energy intake; however, the correlation between age and energy intake was significant (*r* = −0.487, *p* = 0.002) and that of age and protein intake approached significance (*r* = −0.304, *p* = 0.063). Protein intake for 92% of participants met or exceeded the RDA lower limit of 0.8 g·kg^−1^·day^−1^ [2], and 76% were above the alternative recommendation of 1.0 g·kg^−1^·day^−1^ [3]. The contributions of plants, meat, and other animal sources of protein were 37%, 42%, and 21% of total intake, respectively.

**Figure 1:**
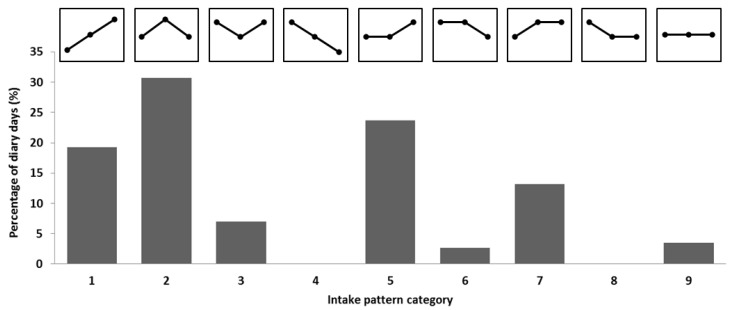
Percentage of days in each category of protein intake pattern. Patterns are depicted above each bar, showing the relationship between periods 1 (05:00–11:00), 2 (11:00–16:00), and 3 (16:00–23:59) in each category.

Distribution of protein was not evenly distributed across the day, as indicated by an average CV for protein distribution of 0.67 (0.20). Periods 1, 2, and 3 included 18%, 39%, and 44% of daily protein, respectively. Main meals were consumed at lunchtime (P2) for 15 participants, with a distribution of 16%:55%:28%, and for the 23 remaining participants dinner (P3) was the main meal, 18%:27%:54%. Categorization of protein intake patterns is illustrated in Figure 1; four categories were highly prevalent (1, 2, 5, and 7), accounting for 86% of the 114 included days. Common to all four of these categories was a small amount of protein at breakfast followed by a relatively larger lunch and/or dinner. The CV for protein distribution did not correlate with protein intake.

**Figure 2:**
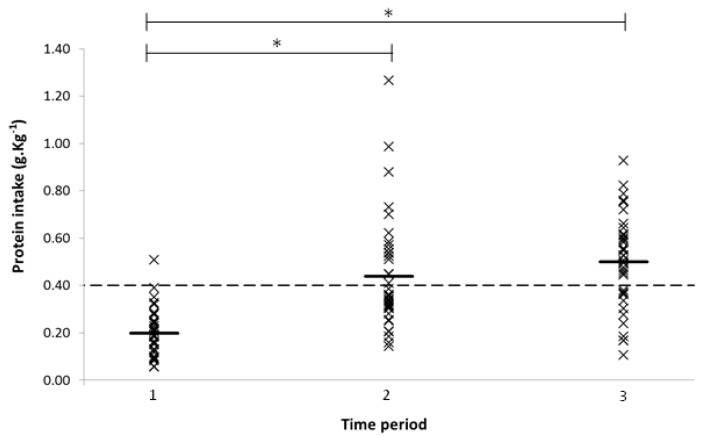
Mean protein intake for each participant by time period (05:00–11:00, 11:00–16:00, 16:00–23:59), bars represent mean for each period. Dashed line represents 0.4 g·kg^−1^ threshold. * indicates significant differences between periods (*p* < 0.05).

When individual meals were compared with the 0.4 g·kg^−1^ threshold for maximal MPS, for time periods 1, 2, and 3, the proportion of participants meeting the threshold was 3%, 42%, and 68% (Figure 2). Further, 8% of participants did not reach the threshold for any of their three daily meals, 71% were below for two meals, and for 21% one of their three meals did not meet the threshold. No participant consumed at least 0.4 g·kg^−1^ in each of their three daily meals. One-way ANOVA comparing total daily protein intake in participants consuming less than the threshold for one, two, and three meals a day indicated a significant difference between groups (*F*(3, 35) = 6.112, *p* = 0.005). Post hoc analysis showed differences between one vs. three meals below (*p* = 0.004) and two vs. three meals below (*p* = 0.015); in both cases, intake was lower in the group consuming three meals per day below the threshold.

### 3.2. Physical Activity and Sedentary Behaviour

Average daily step count was 7136 (3276) steps·day^−1^, and the proportions of stepping, standing and sedentary time were 5%, 13%, and 82% respectively. Step count was significantly correlated with both age (*r* = −0.502, *p* = 0.002) and protein intake (*r* = 0.439, *p* = 0.007).

Daily sedentary time was 18.0 (1.9) h·day^−1^, and sedentary time distribution variables are presented in Table 1. Protein intake was significantly negatively correlated with sedentary time (*r* = −0.456, *p* = 0.005), and with the Gini index (*r* = −0.421, *p* = 0.011), indicating an association between low protein intake and a high volume of sedentary time accumulated in long bouts.

## 4. Discussion

The aim of the current study was to determine total daily protein intake in adults aged over 70 years, and investigate how this is broken down into per meal amounts. We found that the average protein intake in this sample was 1.14 g·kg^−1^·day^−1^. This is a sufficient amount according to recommendations [2,3]; however, per meal intake analysis showed that no participants were consuming 0.4 g·kg^−1^ of protein in all three meals of the day, indicating suboptimal protein delivery according to current literature.

Data from the UK National Diet and Nutrition Survey reports a protein intake of 1.24 g·kg^−1^·day^−1^ for older adults [19], although the analysis included trimming to allow for underreporting, which may account for the lower value in the current study. Analysis from similar Dutch surveys reported intakes of 1.1 g·kg^−1^·day^−1^ and 0.9 g·kg^−1^·day^−1^ in community dwelling adults aged over 65 years [20,21]. Intake was sufficient in all but three participants in our study with respect to the RDA of 0.8–1.2 g·kg^−1^·day^−1^. However, the continuing debate around the protein requirements of older adults has prompted the development of a new recommendation for optimal muscle health, which suggests a higher minimum intake [5]. In light of these more recent recommendations, nine participants had an intake below the suggested limit.

Distribution of protein intake was skewed across the day with particularly small amounts at breakfast, which was consistent with previously reported patterns [20,21]. This was more pronounced when distribution was calculated separately depending on whether the main meal of the day was eaten at lunch or dinner, revealing that over half of the day’s protein was consumed in the main meal. Protein distribution CV was 0.67, also indicating a skewed distribution. A previous study in community-dwelling adults aged ≥75 years reported a higher CV in frail participants compared with non-frail [16], in which a CV of 0.68 for non-frail participants is consistent with our data.

It has been suggested that per meal protein amounts may influence muscle accretion. Cross-sectional data for 50–85 year olds extracted from the 1999–2002 National Health and Nutrition Examination Survey have shown that leg lean mass and strength are associated with the frequency of meals containing at least 30 g of protein [9]. This was based on estimates of a threshold dose for maximal MPS in absolute terms [22,23]; however, our analysis used a proposed threshold expressed relative to body weight [8]. We found that 79% of participants consumed less than the 0.4 g·kg^−1^ threshold in at least two meals per day. Unsurprisingly, total protein intake was significantly lower in the participants who did not reach the threshold for any daily meals, although there was no difference in total protein between the one and two meal groups. No participant consumed three threshold doses a day; to reach three doses of 0.4 g·kg^−1^ in a day there would need to be a slight increase from the average total daily intake. Clearly, there is potential for improvement in terms of protein distribution; however, these data indicate that an increase in total protein intake may be beneficial, to facilitate an optimal distribution of protein doses in line with the acute and cross-sectional data.

Conversely, the two intervention studies which have examined chronic effects of protein distribution in older adults have reported greater improvements in body composition, nitrogen balance, and protein turnover with a skewed distribution compared with an evenly spread intake [24,25]. This may be explained by the protein amounts in the evenly spread distribution, which did not reach 0.4 g·kg^−1^. Interventions to optimize protein distribution should consider per meal protein amounts in relation to data on acute responses.

The basis of this distribution hypothesis is the observation that the relationship between protein dose and MPS response is both dose-dependent and saturable [8,23]; however, the importance of this observation is contentious and not yet fully defined, not least because net muscle accretion is determined by two components of the anabolic response, muscle protein synthesis, and muscle protein breakdown (MPB). Protein ingestion not only stimulates MPS but also prompts a rise in insulin, which suppresses muscle protein breakdown [26], thereby impacting upon both components of the anabolic response. Although the effect of increasing protein dose may plateau in terms of synthesis, there is nothing to suggest that the suppression of breakdown does not continue to increase with higher doses, resulting in a continued net gain [27]. This may negate the potentially beneficial effects on MPS of an even protein distribution in terms of overall muscle protein balance. Due to the challenges associated with MPB measurement, focus in the literature tends towards measurement of only MPS to identify an anabolic response [27]; hence, this response is less well defined, particularly in a chronic setting. Kim et al. [28] included measurements of protein breakdown and net balance in a study of 52–75 years old adults, who consumed either a high or low protein diet divided into an even or uneven distribution for three days. There was no effect of protein distribution on whole body protein synthesis, breakdown or net balance, or on MPS, which would contradict the even distribution hypothesis. However, the results are not consistent with other data showing an effect of distribution on MPS in younger adults [7], nor with reported differences in whole body protein turnover, synthesis, and breakdown in older adults following 14 days of even or uneven protein distribution [24]. Thus, the effects on MPB and net balance of protein distributions that affect MPS are still unclear. This indicates the need for further research into the effects of protein distribution on anabolic responsiveness as a whole, to determine the overall effects on muscle accretion; without further investigation, this remains a limitation of the hypothesis.

Activity data were also included in this study, as both low physical activity and high sedentary time are additional risk factors for sarcopenia [10,11,13], and there are health benefits associated with fragmentation of sedentary time [29]. Average step count was in line with previous results from older adults [30], although 58% of the sample did not meet estimated recommendations [31], and participants spent 75% of their time sedentary. Correlations with protein intake indicated that participants with lower protein intake also had a lower step count and greater sedentary time accumulated in longer bouts. Associations between these risk factors for sarcopenia are worthy of further investigation, as the identification of causal relationships, such as suppressed appetite caused by inactivity potentially resulting in low protein intake, may aid in the development of more effective interventions.

One of the main limitations of this study is the relatively small sample size; however, this was sufficient to detect differences in protein intake. We also did not consider quality of protein with respect to protein thresholds, which can influence the acute MPS response to a dose of protein [32,33].

The strengths of the study include the methods of assessment. ActivPAL™ accelerometry is an objective measure of physical activity that has been validated in terms of step count and active/sedentary time [34,35]. Although not an objective measure, the use of food diaries for dietary assessment in older adults has been validated [36]. Food diary duration has also been addressed; accuracy of three-day records was found to be acceptable when compared with nine-day records in adults aged 40–70 years [37]. Moreover, the allocation of protein to just three meals in a day has previously been considered a limitation [16]. In this analysis, we initially included an additional time period to allow for a snack; however, the protein content of this period was negligible, indicating that it is valid to assess intake in three meals per day. This is the first study to assess per meal protein intake with respect to a threshold protein dose expressed relative to body weight rather than in absolute terms, which is arguably more relevant to dietary recommendations, as they are also given relative to body weight. 

In summary, protein intake was generally aligned with recommendations; however, per meal protein intake relative to body weight was suboptimal according to current literature. Intervention targets may include a focus upon an even distribution to achieve maximal MPS across the day, as well as increased total intake to facilitate optimal distribution. Additionally, lower protein intake was associated with lower physical activity and higher sedentary time, which should be further explored and potentially combined in strategies to reduce the effects of sarcopenia in older adults.

## Figures and Tables

**Table 1:** Participant characteristics and key variable results from dietary analysis and physical activity/sedentary behavior.

**Participant Characteristics**	
*N*	38
Male (*n* (%))	11 (30)
Age (years)	78 (5)
Body weight (kg)	68 (12)
**Dietary Intake**	
Energy intake (kcal·day^−1^)	1815 (363)
Protein intake (g·kg^−1^·day^−1^)	1.14 (0.25)
Protein (energy %)	17.0 (3.4)
**Physical Activity and Sedentary**	
Step count (steps·day^−1^)	7136 (3276)
Sedentary time (h·day^−1^)	18 (1.9)
Standing time (h·day^−1^)	4.5 (1.5)
Active time (h·day^−1^)	1.5 (0.6)
Sedentary bout length (h)	1.6 (0.7)
Inter-sitting time (h)	0.5 (0.1)
Fragmentation index	3.1 (1.0)
Gini index	0.75 (0.04)

Data are presented as mean (SD) unless otherwise stated.

## References

[1] Rosenberg I.H. (1997). Sarcopenia: Origins and clinical relevance. J. Nutr..

[2] Cuthbertson D., Smith K., Babraj J., Leese G., Waddell T., Atherton P., Wackerhage H., Taylor P.M., Rennie M.J. (2005). Anabolic signaling deficits underlie amino acid resistance of wasting, aging muscle. FASEB J..

[3] Greig C.A., Gray C., Rankin D., Young A., Mann V., Noble B., Atherton P.J. (2011). Blunting of adaptive responses to resistance exercise training in women over 75 years. Exp. Gerontol..

[4] Food and Nutrition Board (2005). Protein and amino acids. Dietary Reference Intakes for Energy, Carbohydrate, Fiber, Fat, Fatty Acids, Cholesterol, Protein and Amino Acids (Macronutrients).

[5] Bauer J., Biolo G., Cederholm T., Cesari M., Cruz-Jentoft A.J., Morley J.E., Phillips S., Sieber C., Stehle P., Teta D. (2013). Evidence-based recommendations for optimal dietary protein intake in older people: A position paper from the PROT-AGE Study Group. J. Am. Med. Dir. Assoc..

[6] Areta J.L., Burke L.M., Ross M.L., Camera D.M., West D.W., Broad E.M., Jeacocke N.A., Moore D.R., Stellingwerff T., Phillips S.M. (2013). Timing and distribution of protein ingestion during prolonged recovery from resistance exercise alters myofibrillar protein synthesis. J. Physiol..

[7] Mamerow M.M., Mettler J.A., English K.L., Casperson S.L., Arentson-Lantz E., Sheffield-Moore M., Layman D.K., Paddon-Jones D. (2014). Dietary protein distribution positively influences 24-h muscle protein synthesis in healthy adults. J. Nutr..

[8] Moore D.R., Churchward-Venne T.A., Witard O., Breen L., Burd N.A., Tipton K.D., Phillips S.M. (2015). Protein ingestion to stimulate myofibrillar protein synthesis requires greater relative protein intakes in healthy older versus younger men. J. Gerontol. Ser. A Biol. Sci. Med. Sci..

[9] Loenneke J.P., Loprinzi P.D., Murphy C.H., Phillips S.M. (2016). Per meal dose and frequency of protein consumption is associated with lean mass and muscle performance. Clin. Nutr..

[10] Szulc P., Duboeuf F., Marchand F., Delmas P.D. (2004). Hormonal and lifestyle determinants of appendicular skeletal muscle mass in men: The MINOS study. Am. J. Clin. Nutr..

[11] Little J.P., Phillips S.M. (2009). Resistance exercise and nutrition to counteract muscle wasting. Appl. Physiol. Nutr. Metab..

[12] Scholes S., Mindell J. (2013). Physical activity in adults. Health Survey for England—2012.

[13] Gianoudis J., Bailey C.A., Daly R.M. (2015). Associations between sedentary behaviour and body composition, muscle function and sarcopenia in community-dwelling older adults. Osteoporos. Int..

[14] Physical Activity Guidelines for Older Adults (65+ Years). https://www.nhs.uk/Livewell/fitness/Documents/older-adults-65-years.pdf.

[15] Chastin S.F., Ferriolli E., Stephens N.A., Fearon K.C., Greig C. (2012). Relationship between sedentary behaviour, physical activity, muscle quality and body composition in healthy older adults. Age Ageing.

[16] Bollwein J., Diekmann R., Kaiser M.J., Bauer J.M., Uter W., Sieber C.C., Volkert D. (2013). Distribution but not amount of protein intake is associated with frailty: A cross-sectional investigation in the region of Nürnberg. Nutr. J..

[17] Chastin S.F., Granat M.H. (2010). Methods for objective measure, quantification and analysis of sedentary behaviour and inactivity. Gait Posture.

[18] Tieges Z., Mead G., Allerhand M., Duncan F., van Wijck F., Fitzsimons C., Greig C., Chastin S. (2015). Sedentary behavior in the first year after stroke: A longitudinal cohort study with objective measures. Arch. Phys. Med. Rehabil..

[19] Millward D.J. (2012). Nutrition and sarcopenia: Evidence for an interaction. Proc. Nutr. Soc..

[20] Tieland M., Borgonjen-Van den Berg K.J., van Loon L.J., de Groot L.C. (2015). Dietary protein intake in Dutch elderly people: A focus on protein sources. Nutrients.

[21] Tieland M., Borgonjen-Van den Berg K.J., van Loon L.J., de Groot L.C. (2012). Dietary protein intake in community-dwelling, frail, and institutionalized elderly people: Scope for improvement. Eur. J. Nutr..

[22] Symons T.B., Sheffield-Moore M., Wolfe R.R., Paddon-Jones D. (2009). A moderate serving of high-quality protein maximally stimulates skeletal muscle protein synthesis in young and elderly subjects. J. Am. Diet. Assoc..

[23] Yang Y., Breen L., Burd N.A., Hector A.J., Churchward-Venne T.A., Josse A.R., Tarnopolsky M.A., Phillips S.M. (2012). Resistance exercise enhances myofibrillar protein synthesis with graded intakes of whey protein in older men. Br. J. Nutr..

[24] Arnal M., Mosoni L., Boirie Y., Houlier M., Morin L., Verdier E., Ritz P., Antoine J., Prugnaud J., Beaufrere B. (1999). Protein pulse feeding improves protein retention in elderly women. Am. J. Clin. Nutr..

[25] Bouillanne O., Curis E., Hamon-Vilcot B., Nicolis I., Chretien P., Schauer N., Vincent J.P., Cynober L., Aussel C. (2013). Impact of protein pulse feeding on lean mass in malnourished and at-risk hospitalized elderly patients: A randomized controlled trial. Clin. Nutr..

[26] Greenhaff P.L., Karagounis L.G., Peirce N., Simpson E.J., Hazell M., Layfield R., Wackerhage H., Smith K., Atherton P., Selby A. (2008). Disassociation between the effects of amino acids and insulin on signaling, ubiquitin ligases, and protein turnover in human muscle. Am. J. Physiol. Endocrinol. Metab..

[27] Deutz N.E., Wolfe R.R. (2013). Is there a maximal anabolic response to protein intake with a meal?. Clin. Nutr..

[28] Kim I.Y., Schutzler S., Schrader A., Spencer H., Kortebein P., Deutz N.E., Wolfe R.R., Ferrando A.A. (2015). Quantity of dietary protein intake, but not pattern of intake, affects net protein balance primarily through differences in protein synthesis in older adults. Am. J. Physiol. Endocrinol. Metab..

[29] Healy G.N., Dunstan D.W., Salmon J., Cerin E., Shaw J.E., Zimmet P.Z., Owen N. (2008). Breaks in sedentary time: Beneficial associations with metabolic risk. Diabetes Care.

[30] Tudor-Locke C.E., Myers A.M. (2001). Methodological considerations for researchers and practitioners using pedometers to measure physical (ambulatory) activity. Res. Quart. Exerc. Sport.

[31] Tudor-Locke C., Craig C.L., Aoyagi Y., Bell R.C., Croteau K.A., de Bourdeaudhuij I., Ewald B., Gardner A.W., Hatano Y., Lutes L.D. (2011). How many steps/day are enough? For older adults and special populations. Int. J. Behav. Nutr. Phys. Activ..

[32] Phillips S.M., Tang J.E., Moore D.R. (2009). The role of milk- and soy-based protein in support of muscle protein synthesis and muscle protein accretion in young and elderly persons. J. Am. Coll. Nutr..

[33] Van Vliet S., Burd N.A., van Loon L.J. (2015). The skeletal muscle anabolic response to plant-versus animal-based protein consumption. J. Nutr..

[34] Grant P.M., Ryan C.G., Tigbe W.W., Granat M.H. (2006). The validation of a novel activity monitor in the measurement of posture and motion during everyday activities. Br. J. Sports Med..

[35] Grant P.M., Dall P.M., Mitchell S.L., Granat M.H. (2008). Activity-monitor accuracy in measuring step number and cadence in community-dwelling older adults. J. Aging Phys. Activ..

[36] Luhrmann P.M., Herbert B.M., Gaster C., Neuhauser-Berthold M. (1999). Validation of a self-administered 3-day estimated dietary record for use in the elderly. Eur. J. Nutr..

[37] Yang Y.J., Kim M.K., Hwang S.H., Ahn Y., Shim J.E., Kim D.H. (2010). Relative validities of 3-day food records and the food frequency questionnaire. Nutr. Res. Pract..

